# Structure of Equilenin at 100 K: an estrone-related steroid

**DOI:** 10.1107/S2056989017010532

**Published:** 2017-07-21

**Authors:** Christopher S. Frampton, David D. MacNicol

**Affiliations:** a Wolfson Centre for Materials Processing, Brunel University London, Kingston Lane, Uxbridge UB8 3PH, England; b Department of Chemistry, University of Glasgow, Glasgow G12 8QQ, Scotland

**Keywords:** crystal structure, Equilenin, Equilin, estrone, steroid, conformation, hydrogen bonding

## Abstract

The structure of the estrone-related steroid, Equilenin, C_18_H_18_O_2_ (systematic name 3-hy­droxy-13-methyl-11,12,13,14,15,16-hexa­hydro­cyclo­penta­[*a*]phen­anthren-17-one), has been determined at 100 K. The crystals are ortho­rhom­bic, *P*2_1_2_1_2_1_, and the absolute structure of the mol­ecule in the crystal has been determined by resonant scattering [Flack parameter = −0.05 (4)]. The C atoms of the *A* and *B* rings are almost coplanar, with an r.m.s. deviation from planarity of 0.0104 Å. The *C* ring has a sofa conformation, while the *D* ring has an envelope conformation with the methine C atom as the flap. The keto O atom and the methyl group are translated 0.78 and 0.79 Å, respectively, from the equivalent positions on 17β-estrone. In the crystal, mol­ecules are linked by O—H⋯O hydrogen bonds, forming chains parallel to the *c*-axis direction.

## Abstract 2

The structure of the estrone related steroid, Equilenin **1**, has been determined at 100 K. It is of great inter­est to investigate what the structural and conformational consequences are on the *C* and *D* rings of the steroid framework of **1** by having fully unsaturated *A* and *B* rings.

## Chemical context   

The title compound, Equilenin **1**, is one member of a series of three estrogenic steroids, the other members being Equilin **2** and 17β-estrone **3**, that are components of the hormone replacement therapy medication, ‘*Premarin*’, a mixture of natural estrogens isolated from the urine of pregnant equine mares. It can be seen from the scheme that on going from 17β-estrone **3** through to the title compound Equilenin **1**, there is a progressive aromatization of the *B* ring of the steroid framework where in **1** rings *A* and *B* comprise a fully aromatic naphthalene core. The structure of Equilin **2**, was determined by Sawicki *et al.* (1999*b* ▸), who demonstrated that the presence of the unsaturated C7—C8 bond in the *B* ring rotates the *C* and *D* rings of the steroid such that the 17-keto oxygen atom, O17, is translated by 0.73 Å with respect to the analogous oxygen atom of **3** when an overlay of the two structures was performed based on the superposition of the *A* rings. The translation of the oxygen atom was implicated in the increased anti-human estrogenic 17β-hy­droxy­steroid de­hydrogenase 1 (17β-HSD1) inhibitory behaviour of **2** with respect to 17β-estone **3** (Sawicki *et al.*, 1999*a* ▸). The impact of the inhibitory behaviour of **2** is that it causes a reduction of the active estrogen, 17β-estradiol, which is present in elevated concentrations in human breast tumour tissues and responsible for the accelerated growth of the tumour tissue. It is therefore of great inter­est to investigate what the structural and conformational consequences are on the *C* and *D* rings of the steroid framework of **1** by having fully unsaturated *A* and *B* rings. Although the unit-cell parameters of **1** at room temperature have been previously reported by Ohrt *et al.* (1967 ▸), no three-dimensional structure analysis of this important estrone steroid has been determined. Herein, we report on the crystal structure of this final member of the estrone series of steroids, Equilenin **1**, at 100 K.
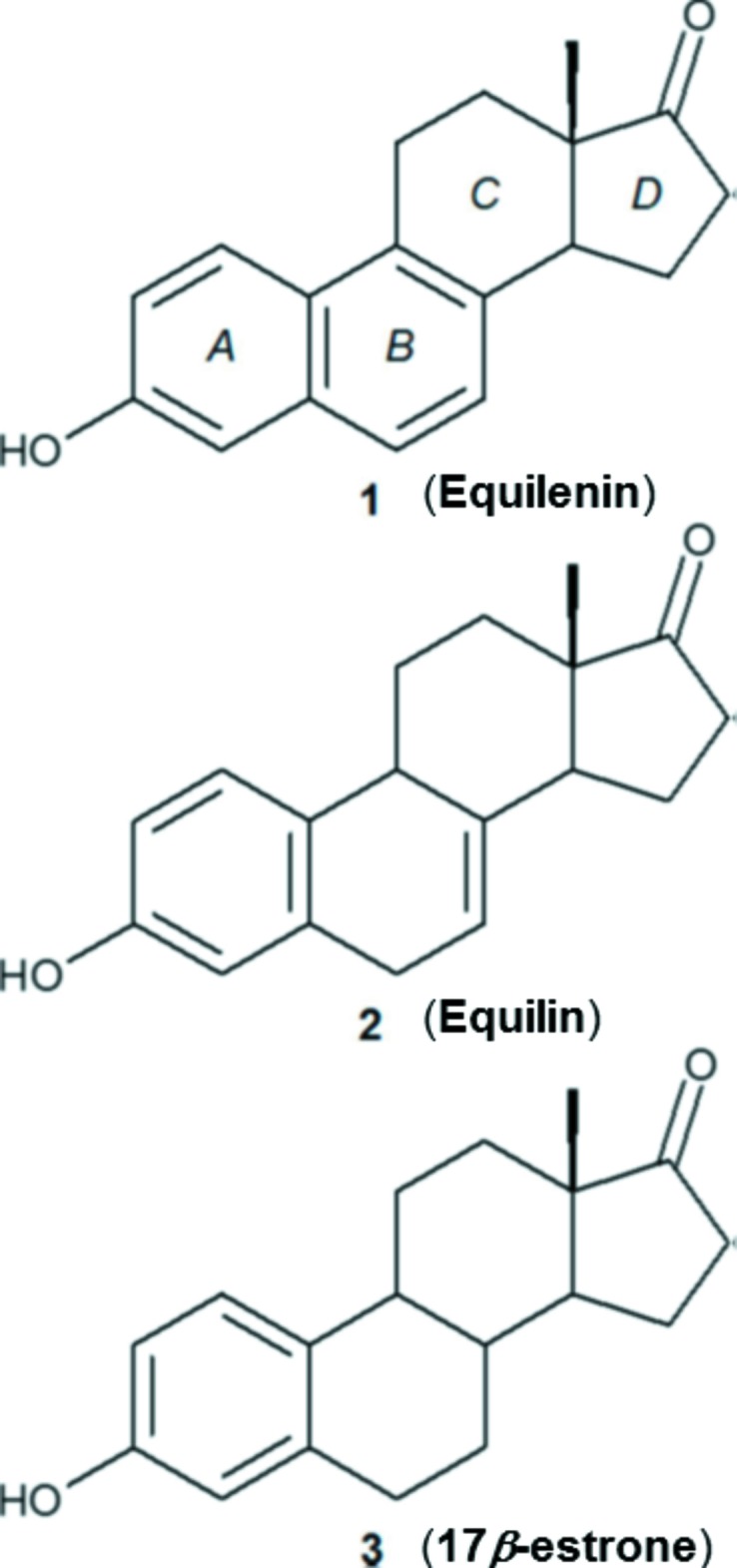


## Structural commentary   

**Figure 1:**
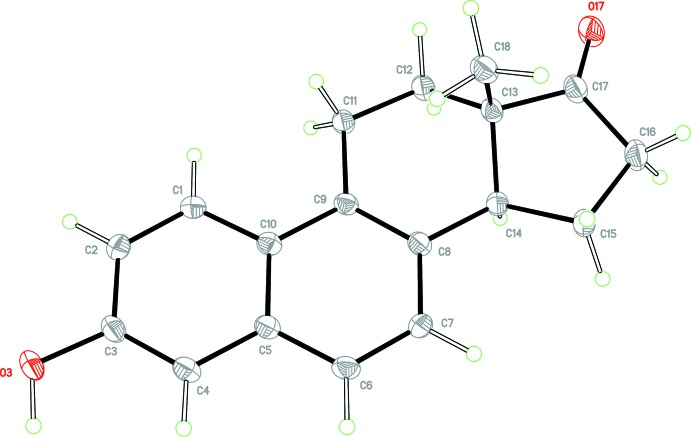
View of the mol­ecular structure of compound **1**, with the atom labelling. Displacement ellipsoids are drawn at the 50% probability level.

The crystal structure of Equilenin **1**, is ortho­rhom­bic, space group *P*2_1_2_1_2_1_ (*Z* ’= 1) and its mol­ecular structure is illustrated in Fig. 1 ▸. The unit cell data agree with the previously reported values (Ohrt *et al.*, 1967 ▸) with the caveat that they are slightly smaller owing to some modest isotropic contraction due to the lower temperature. The atoms C1 through C10, which define the *AB* (napthalene) plane, are little affected by the chiral centres at C13 and C14, and are almost coplanar with an r.m.s. deviation of the fitted atoms of 0.0104 Å and a total puckering amplitude, *Q*, of 0.033 (2) Å. The greatest displacement from the ten-atom mean plane is atom C10 at −0.019 (1) Å. The C—C bond lengths of the *AB* rings follow the pattern in which C1—C2, C3—C4, C6—C7 and C8—C9 are significantly shorter, (mean value 1.372 Å), than the remaining seven bonds (mean value 1.421 Å) [Ahmed & Cruickshank, 1952 ▸; Cruickshank & Sparks, 1960 ▸], thus demonstrating that the *AB* ring system is a true aromatic naphthalene core. The aromatization of ring *B* does however, have a significant effect on the conformations of both the *C* and *D* rings of **1**, compared to **2** and **3.** In contrast to the regular chair conformation of the *C* rings of **2** and **3**, the *C* ring of **1**, has a highly symmetric 13β-envelope conformation characterized by a Δ*C*_s_(9) asymmetry parameter of 0.50° (Duax *et al.*, 1976 ▸); and related pairs of torsion angles [C14—C8—C9—C11, C8—C9—C11—C12, −4.1 (2), 4.1 (2)°; C9—C11—C12—C13, C9—C8—C14—C13, −32.6 (2), 32.7 (2)°; C11—C12—C13—C14, C12—C13—C14—C8, 60.4 (2), −61.3 (1)°]. The downside impact of this conformational change in the *C* ring of **1** is such that in place of the asymmetric twist or half-chair *D* ring conformation demonstrated by **2** and **3**, the *D* ring of Equilenin **1** displays a 14α-envelope conformation with a ΔC_s_(14) of 4.20°; the torsion angles for **1**, (with related torsion angles for **2**/**3** are given in [/]) are C13—C14—C15—C16, C17—C13—C14—C15) −41.3 (2) [−40.2/-39.0]°, 43.3 (2) [44.5/42.9]°; C14—C13—C17—C16, C14—C15—C16—C17, −28.6 (2) [−31.0/-30.9]°, 23.0 (2) [19.6/19.4]°; and C15—C16—C17—C13, 3.6 (2) [8.1/7.5]°. Torsional angle data for **2** and **3** were extracted from structures GODTIC (Sawicki *et al.*, 1999*b* ▸) and ESTRON13 (Shikii *et al.*, 2004 ▸), respectively (see Section 4, *Database survey*).

**Figure 2:**
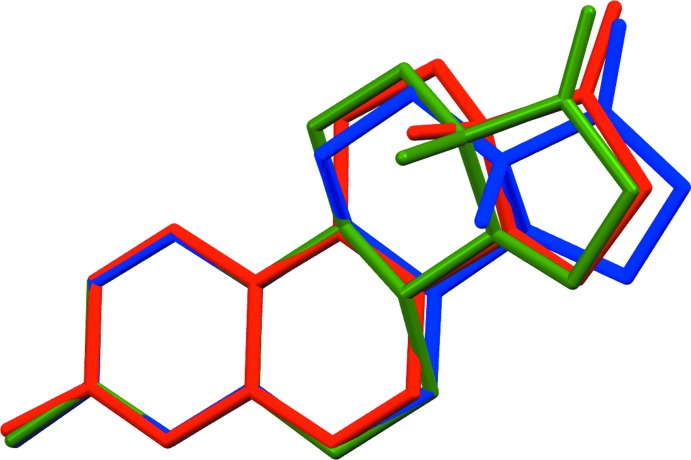
View of the structure overlay of compounds **1** (red), **2** (blue) and **3** (green). The overlay was performed by a superposition of the atoms in the *A* ring only.

Compounds **1** and **2**, possibly owing to increased conformational constraint in the *B* ring, have lower oestrongenic activity than 17β-estrone itself, which has the *B* ring as the principal point of mol­ecular flexibility (Duax *et al.*, 1976 ▸; Busetta *et al.*, 1973 ▸). Inter­estingly this reduction in activity (Marshall, 1970 ▸) does not directly relate to the crystallographically determined degree to which the *A* and *B* rings of the steroid are constrained to coplanarity, since **1**, possessing an essentially planar naphthalene core, is about five times more estrogenic than **2** which features only approximate coplanarity of its *A* and *B* rings with an r.m.s. deviation of the fitted atoms of 0.102 Å, and a total puckering amplitude of 0.270 (2) Å (Sawicki *et al.*, 1999*b* ▸). An overlay of structures **1** (red), **2** (blue) and **3** (green) is shown in Fig. 2 ▸. The overlay was performed by a superposition of the atoms in the *A* ring only. From this overlay it can be calculated that the keto oxygen atom is translated by 0.78 and 0.69 Å, respectively, for compounds **1** and **2** from its position on **3**. Perhaps more significant is the degree of translation of the methyl group C18 which is translated by 0.79 and 1.40 Å, respectively, for compounds **1** and **2** from its position on **3** which may account for the increased estrogenic activity of **1** over **2**. The stereochemistry assignments at C13 and C14 are *S*, *S*; confirmed by resonant scattering through the Flack *x* parameter value of −0.05 (4).

## Supra­molecular features   

**Figure 3:**
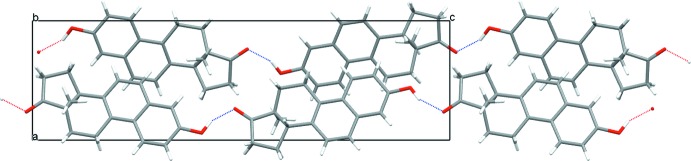
View along the *b* axis of the crystal packing of compound **1**. The inter­molecular O—H⋯O hydrogen bonds are shown as dashed lines (see Table 1 ▸).

In the crystal, the Equilenin **1** mol­ecules are linked head-to-tail by a single O—H⋯O^i^ hydrogen bond (Table 1 ▸), to form chains propagating along the *c*-axis direction. A view along the *b*-axis of the crystal packing of the title compound is shown in Fig. 3 ▸.

## Database survey   

A search of the Cambridge Structural Database (CSD, Version 5.38, last update February 2017; Groom *et al.*, 2016 ▸) for the basic steroid *ABCD* ring framework yielded 401 hits although the hits for **1** and **2** could only be accessed by introducing the aromaticity into the *B* ring. Of the 401 hits there were eight hits for the structure of 17β-estrone, **3** (ESTRON03–05 and ESTRON10–15), which exists in three polymorphic forms. They include, form I, ortho­rhom­bic *P*2_1_2_1_2_1_ (ESTRON11: Busetta *et al.*, 1973 ▸), form II, ortho­rhom­bic *P*2_1_2_1_2_1_ (ESTRON03: Debaerdemaeker, 1972 ▸; ESTRON04: van den Bossche *et al.*, 1971 ▸; ESTRON10: Busetta *et al.*, 1973 ▸; ESTRON13: Shikii *et al.*, 2004 ▸; ESTRON14: Zhurova *et al.*, 2006 ▸) and form III, monoclinic *P*2_1_ (*Z′* = 2) (ESTRON05, unit-cell determination only: Ohrt *et al.*, 1964 ▸; ESTRON12: Busetta *et al.*, 1973 ▸). The polymorphic behaviour appears to be attributable to the crystal packing and has no significant influence on the conformation of the steroid framework. There was a single entry for **2** (GODTIC: Sawicki *et al.*, 1999*b* ▸) and a single entry for **1** (QQQAMM, unit-cell determination only: Ohrt *et al.*, 1967 ▸).

## Synthesis and crystallization   

In common with Equilin **2** the title compound, **1**, was isolated from the urine of a pregnant mare (Girard *et al.*, 1932 ▸; Fieser & Fieser, 1959 ▸). The sample used for the X-ray data collection was gifted to us from the J. W. Cook collection of the University of Glasgow. Suitable crystals were obtained as needles from ethanol solution, m.p. 531–532 K (evacuated sealed capillary).

## Refinement   

Crystal data, data collection and structure refinement details are summarized in Table 2 ▸. The O-bound H atom was located from a difference-Fourier map and freely refined. All remaining H atoms were placed geometrically in idealized positions and refined using a riding model (including free rotation about the methyl C—C bond): C—H = 0.95–0.99 Å with *U*_iso_(H) = 1.5*U*_eq_(C-meth­yl) and 1.2*U*_eq_(C) for other H atoms. The absolute stereochemistry of **1**, was confirmed through the Flack *x* parameter value of −0.05 (4). This was determined using 1130 quotients [(*I* ^+^)−(*I* ^−^)]/[(*I* ^+^)+(*I* ^−^)] (Parsons *et al.*, 2013 ▸).

## Supplementary Material

Crystal structure: contains datablock(s) I. DOI: 10.1107/S2056989017010532/su5383sup1.cif

Structure factors: contains datablock(s) I. DOI: 10.1107/S2056989017010532/su5383Isup2.hkl

Supporting information file. DOI: 10.1107/S2056989017010532/su5383Isup3.cml

CCDC reference: 1560649

Additional supporting information:  crystallographic information; 3D view; checkCIF report

## Figures and Tables

**Table 1:** Hydrogen-bond geometry (Å, °)

*D*—H⋯*A*	*D*—H	H⋯*A*	*D*⋯*A*	*D*—H⋯*A*
O3—H3*A*⋯O17^i^	0.95 (3)	1.82 (3)	2.7153 (17)	157 (3)

Symmetry code: (i) 
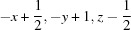
.

**Table 2:** Experimental details

Crystal data	
Chemical formula	C_18_H_18_O_2_
*M*_r_	266.32
Crystal system, space group	Orthorhombic, *P*2_1_2_1_2_1_
Temperature (K)	100
*a*, *b*, *c* (Å)	7.27709 (7), 7.32686 (6), 25.5179 (2)
*V* (Å^3^)	1360.57 (2)
*Z*	4
Radiation type	Cu *K*α
μ (mm^−1^)	0.66
Crystal size (mm)	0.41 × 0.12 × 0.07

Data collection	
Diffractometer	Rigaku Oxford Diffraction SuperNova Dualflex AtlasS2
Absorption correction	Multi-scan (*CrysAlis PRO*; Rigaku Oxford Diffraction, 2015 ▸)
*T*_min_, *T*_max_	0.853, 0.960
No. of measured, independent and observed [*I* > 2σ(*I*)] reflections	16660, 2769, 2754
*R*_int_	0.022
(sin θ/λ)_max_ (Å^−1^)	0.625

Refinement	
*R*[*F* ^2^ > 2σ(*F* ^2^)], *wR*(*F* ^2^), *S*	0.029, 0.080, 1.01
No. of reflections	2769
No. of parameters	186
H-atom treatment	H atoms treated by a mixture of independent and constrained refinement
Δρ_max_, Δρ_min_ (e Å^−3^)	0.26, −0.17
Absolute structure	Flack *x* determined using 1130 quotients [(*I* ^+^)−(*I* ^−^)]/[(*I* ^+^)+(*I* ^−^)] (Parsons *et al.*, 2013 ▸)
Absolute structure parameter	−0.05 (4)

Computer programs: *CrysAlis PRO* (Rigaku Oxford Diffraction, 2015 ▸), *SHELXD2014* (Sheldrick, 2010 ▸), *SHELXL2014* (Sheldrick, 2015 ▸), *SHELXTL* (Sheldrick, 2008 ▸) and *Mercury* (Macrae *et al.*, 2008 ▸), *SHELXTL* (Sheldrick, 2008 ▸) and *publCIF* (Westrip, 2010 ▸).

## supplementary crystallographic information

### Crystal data

**Table:**

C_18_H_18_O_2_	*D*_x_ = 1.300 Mg m^−^^3^
*M**_r_* = 266.32	Melting point: 531 K
Orthorhombic, *P*2_1_2_1_2_1_	Cu *K*α radiation, λ = 1.54184 Å
*a* = 7.27709 (7) Å	Cell parameters from 13177 reflections
*b* = 7.32686 (6) Å	θ = 3.5–76.4°
*c* = 25.5179 (2) Å	µ = 0.66 mm^−^^1^
*V* = 1360.57 (2) Å^3^	*T* = 100 K
*Z* = 4	Rod, colourless
*F*(000) = 568	0.41 × 0.12 × 0.07 mm

### Data collection

**Table:**

Rigaku Oxford Diffraction SuperNova Dualflex AtlasS2 diffractometer	2769 independent reflections
Radiation source: fine-focus sealed X-ray tube, Enhance (Cu) X-ray Source	2754 reflections with *I* > 2σ(*I*)
Detector resolution: 5.2921 pixels mm^-1^	*R*_int_ = 0.022
ω scans	θ_max_ = 74.5°, θ_min_ = 3.5°
Absorption correction: multi-scan (CrysAlis PRO; Rigaku Oxford Diffraction, 2015)	*h* = −9→7
*T*_min_ = 0.853, *T*_max_ = 0.960	*k* = −9→9
16660 measured reflections	*l* = −31→31

### Refinement

**Table:**

Refinement on *F*^2^	Secondary atom site location: difference Fourier map
Least-squares matrix: full	Hydrogen site location: mixed
*R*[*F*^2^ > 2σ(*F*^2^)] = 0.029	H atoms treated by a mixture of independent and constrained refinement
*wR*(*F*^2^) = 0.080	*w* = 1/[σ^2^(*F*_o_^2^) + (0.0525*P*)^2^ + 0.310*P*] where *P* = (*F*_o_^2^ + 2*F*_c_^2^)/3
*S* = 1.01	(Δ/σ)_max_ < 0.001
2769 reflections	Δρ_max_ = 0.26 e Å^−^^3^
186 parameters	Δρ_min_ = −0.17 e Å^−^^3^
0 restraints	Absolute structure: Flack *x* determined using 1130 quotients [(*I*^+^)-(*I*^-^)]/[(*I*^+^)+(*I*^-^)] (Parsons *et al.*, 2013)
Primary atom site location: structure-invariant direct methods	Absolute structure parameter: −0.05 (4)

### Special details

**Table:**

Geometry. All esds (except the esd in the dihedral angle between two l.s. planes) are estimated using the full covariance matrix. The cell esds are taken into account individually in the estimation of esds in distances, angles and torsion angles; correlations between esds in cell parameters are only used when they are defined by crystal symmetry. An approximate (isotropic) treatment of cell esds is used for estimating esds involving l.s. planes.

### Fractional atomic coordinates and isotropic or equivalent isotropic displacement parameters (Å^2^)

**Table:**

	*x*	*y*	*z*	*U*_iso_*/*U*_eq_	
O3	0.06030 (17)	0.42177 (17)	0.09478 (4)	0.0234 (3)	
H3A	0.155 (4)	0.472 (4)	0.0738 (10)	0.058 (8)*	
O17	0.23720 (17)	0.44067 (19)	0.51461 (4)	0.0277 (3)	
C1	−0.0140 (2)	0.4004 (2)	0.23492 (6)	0.0184 (3)	
H1A	−0.1120	0.3698	0.2579	0.022*	
C2	−0.0417 (2)	0.3930 (2)	0.18189 (6)	0.0191 (3)	
H2A	−0.1585	0.3588	0.1685	0.023*	
C3	0.1025 (2)	0.4357 (2)	0.14700 (6)	0.0184 (3)	
C4	0.2710 (2)	0.4870 (2)	0.16578 (6)	0.0185 (3)	
H4A	0.3673	0.5152	0.1420	0.022*	
C5	0.3030 (2)	0.4983 (2)	0.22075 (6)	0.0164 (3)	
C6	0.4744 (2)	0.5542 (2)	0.24123 (6)	0.0191 (3)	
H6A	0.5719	0.5844	0.2181	0.023*	
C7	0.5009 (2)	0.5651 (2)	0.29424 (6)	0.0182 (3)	
H7A	0.6172	0.6022	0.3073	0.022*	
C8	0.3584 (2)	0.5223 (2)	0.32991 (6)	0.0162 (3)	
C9	0.1889 (2)	0.4662 (2)	0.31167 (6)	0.0156 (3)	
C10	0.1585 (2)	0.4530 (2)	0.25640 (6)	0.0156 (3)	
C11	0.0307 (2)	0.4177 (2)	0.34819 (6)	0.0181 (3)	
H11A	−0.0010	0.2877	0.3427	0.022*	
H11B	−0.0777	0.4910	0.3379	0.022*	
C12	0.0654 (2)	0.4477 (2)	0.40723 (6)	0.0188 (3)	
H12A	0.0321	0.5743	0.4169	0.023*	
H12B	−0.0125	0.3634	0.4279	0.023*	
C13	0.2666 (2)	0.4136 (2)	0.41985 (5)	0.0174 (3)	
C14	0.3840 (2)	0.5486 (2)	0.38816 (6)	0.0175 (3)	
H14A	0.3364	0.6733	0.3965	0.021*	
C15	0.5749 (2)	0.5349 (3)	0.41347 (6)	0.0233 (3)	
H15A	0.6510	0.6432	0.4053	0.028*	
H15B	0.6404	0.4234	0.4020	0.028*	
C16	0.5263 (2)	0.5265 (3)	0.47245 (6)	0.0278 (4)	
H16A	0.6101	0.4423	0.4911	0.033*	
H16B	0.5360	0.6491	0.4886	0.033*	
C17	0.3293 (2)	0.4572 (2)	0.47493 (6)	0.0210 (3)	
C18	0.3214 (2)	0.2130 (2)	0.41011 (6)	0.0209 (3)	
H18A	0.2300	0.1321	0.4261	0.031*	
H18B	0.4422	0.1896	0.4257	0.031*	
H18C	0.3269	0.1899	0.3723	0.031*	

### Atomic displacement parameters (Å^2^)

**Table:**

	*U*^11^	*U*^22^	*U*^33^	*U*^12^	*U*^13^	*U*^23^
O3	0.0254 (6)	0.0327 (6)	0.0121 (5)	−0.0031 (5)	−0.0025 (4)	0.0006 (4)
O17	0.0239 (6)	0.0460 (7)	0.0131 (5)	−0.0037 (6)	0.0025 (4)	−0.0009 (5)
C1	0.0173 (7)	0.0209 (7)	0.0170 (7)	−0.0030 (6)	0.0015 (6)	0.0020 (5)
C2	0.0163 (7)	0.0216 (7)	0.0193 (7)	−0.0032 (6)	−0.0028 (6)	0.0011 (6)
C3	0.0219 (7)	0.0202 (7)	0.0132 (7)	0.0004 (6)	−0.0015 (6)	0.0014 (6)
C4	0.0191 (7)	0.0218 (7)	0.0146 (7)	0.0019 (6)	0.0025 (6)	0.0023 (5)
C5	0.0160 (7)	0.0183 (7)	0.0149 (7)	0.0011 (6)	0.0011 (5)	0.0020 (5)
C6	0.0146 (7)	0.0254 (8)	0.0171 (7)	0.0000 (6)	0.0037 (6)	0.0035 (6)
C7	0.0122 (7)	0.0243 (8)	0.0182 (7)	−0.0020 (6)	−0.0012 (6)	0.0030 (6)
C8	0.0157 (7)	0.0192 (7)	0.0136 (7)	0.0012 (6)	0.0005 (5)	0.0017 (6)
C9	0.0145 (7)	0.0183 (7)	0.0141 (6)	0.0004 (6)	0.0020 (5)	0.0019 (5)
C10	0.0153 (7)	0.0163 (7)	0.0152 (7)	0.0014 (6)	0.0012 (5)	0.0014 (5)
C11	0.0132 (7)	0.0269 (8)	0.0142 (7)	−0.0015 (6)	0.0011 (5)	0.0009 (6)
C12	0.0132 (7)	0.0291 (8)	0.0143 (6)	0.0007 (6)	0.0021 (5)	0.0000 (6)
C13	0.0146 (7)	0.0256 (7)	0.0121 (6)	0.0002 (6)	0.0006 (5)	0.0010 (6)
C14	0.0145 (7)	0.0241 (7)	0.0140 (7)	−0.0019 (6)	−0.0004 (5)	0.0004 (6)
C15	0.0154 (7)	0.0393 (9)	0.0153 (7)	−0.0042 (7)	−0.0021 (5)	0.0029 (7)
C16	0.0200 (8)	0.0483 (10)	0.0151 (7)	−0.0047 (8)	−0.0027 (6)	0.0019 (7)
C17	0.0193 (7)	0.0287 (8)	0.0148 (7)	0.0018 (7)	−0.0010 (5)	0.0011 (6)
C18	0.0195 (7)	0.0261 (8)	0.0170 (7)	0.0013 (6)	0.0010 (6)	0.0030 (6)

### Geometric parameters (Å, º)

**Table:**

O3—C3	1.3714 (17)	C11—C12	1.5433 (19)
O3—H3A	0.95 (3)	C11—H11A	0.9900
O17—C17	1.220 (2)	C11—H11B	0.9900
C1—C2	1.369 (2)	C12—C13	1.520 (2)
C1—C10	1.423 (2)	C12—H12A	0.9900
C1—H1A	0.9500	C12—H12B	0.9900
C2—C3	1.411 (2)	C13—C17	1.5118 (19)
C2—H2A	0.9500	C13—C14	1.537 (2)
C3—C4	1.369 (2)	C13—C18	1.544 (2)
C4—C5	1.4243 (19)	C14—C15	1.535 (2)
C4—H4A	0.9500	C14—H14A	1.0000
C5—C6	1.413 (2)	C15—C16	1.547 (2)
C5—C10	1.429 (2)	C15—H15A	0.9900
C6—C7	1.369 (2)	C15—H15B	0.9900
C6—H6A	0.9500	C16—C17	1.523 (2)
C7—C8	1.414 (2)	C16—H16A	0.9900
C7—H7A	0.9500	C16—H16B	0.9900
C8—C9	1.381 (2)	C18—H18A	0.9800
C8—C14	1.5103 (19)	C18—H18B	0.9800
C9—C10	1.4309 (19)	C18—H18C	0.9800
C9—C11	1.5234 (19)		
			
C3—O3—H3A	111.0 (17)	C13—C12—H12A	109.7
C2—C1—C10	121.43 (14)	C11—C12—H12A	109.7
C2—C1—H1A	119.3	C13—C12—H12B	109.7
C10—C1—H1A	119.3	C11—C12—H12B	109.7
C1—C2—C3	120.34 (14)	H12A—C12—H12B	108.2
C1—C2—H2A	119.8	C17—C13—C12	116.95 (12)
C3—C2—H2A	119.8	C17—C13—C14	100.69 (12)
C4—C3—O3	124.14 (14)	C12—C13—C14	108.58 (12)
C4—C3—C2	120.39 (13)	C17—C13—C18	105.82 (13)
O3—C3—C2	115.46 (14)	C12—C13—C18	111.80 (13)
C3—C4—C5	120.47 (14)	C14—C13—C18	112.60 (12)
C3—C4—H4A	119.8	C8—C14—C15	121.14 (13)
C5—C4—H4A	119.8	C8—C14—C13	111.54 (13)
C6—C5—C4	121.68 (14)	C15—C14—C13	103.87 (12)
C6—C5—C10	118.77 (13)	C8—C14—H14A	106.5
C4—C5—C10	119.55 (14)	C15—C14—H14A	106.5
C7—C6—C5	120.43 (14)	C13—C14—H14A	106.5
C7—C6—H6A	119.8	C14—C15—C16	101.83 (12)
C5—C6—H6A	119.8	C14—C15—H15A	111.4
C6—C7—C8	121.33 (14)	C16—C15—H15A	111.4
C6—C7—H7A	119.3	C14—C15—H15B	111.4
C8—C7—H7A	119.3	C16—C15—H15B	111.4
C9—C8—C7	120.24 (13)	H15A—C15—H15B	109.3
C9—C8—C14	118.66 (13)	C17—C16—C15	105.59 (13)
C7—C8—C14	120.98 (14)	C17—C16—H16A	110.6
C8—C9—C10	119.37 (13)	C15—C16—H16A	110.6
C8—C9—C11	122.58 (13)	C17—C16—H16B	110.6
C10—C9—C11	118.06 (13)	C15—C16—H16B	110.6
C1—C10—C5	117.81 (13)	H16A—C16—H16B	108.8
C1—C10—C9	122.30 (13)	O17—C17—C13	125.78 (15)
C5—C10—C9	119.86 (14)	O17—C17—C16	125.78 (15)
C9—C11—C12	116.13 (13)	C13—C17—C16	108.43 (13)
C9—C11—H11A	108.3	C13—C18—H18A	109.5
C12—C11—H11A	108.3	C13—C18—H18B	109.5
C9—C11—H11B	108.3	H18A—C18—H18B	109.5
C12—C11—H11B	108.3	C13—C18—H18C	109.5
H11A—C11—H11B	107.4	H18A—C18—H18C	109.5
C13—C12—C11	109.95 (12)	H18B—C18—H18C	109.5
			
C10—C1—C2—C3	0.7 (2)	C10—C9—C11—C12	−175.62 (14)
C1—C2—C3—C4	−0.7 (2)	C9—C11—C12—C13	−32.63 (19)
C1—C2—C3—O3	179.27 (14)	C11—C12—C13—C17	173.40 (14)
O3—C3—C4—C5	179.87 (14)	C11—C12—C13—C14	60.42 (17)
C2—C3—C4—C5	−0.2 (2)	C11—C12—C13—C18	−64.41 (16)
C3—C4—C5—C6	−178.75 (15)	C9—C8—C14—C15	155.31 (15)
C3—C4—C5—C10	1.0 (2)	C7—C8—C14—C15	−28.7 (2)
C4—C5—C6—C7	179.52 (14)	C9—C8—C14—C13	32.65 (19)
C10—C5—C6—C7	−0.2 (2)	C7—C8—C14—C13	−151.31 (14)
C5—C6—C7—C8	−0.4 (2)	C17—C13—C14—C8	175.33 (13)
C6—C7—C8—C9	0.7 (2)	C12—C13—C14—C8	−61.31 (16)
C6—C7—C8—C14	−175.24 (15)	C18—C13—C14—C8	63.05 (16)
C7—C8—C9—C10	−0.4 (2)	C17—C13—C14—C15	43.25 (15)
C14—C8—C9—C10	175.63 (14)	C12—C13—C14—C15	166.61 (13)
C7—C8—C9—C11	179.82 (14)	C18—C13—C14—C15	−69.03 (16)
C14—C8—C9—C11	−4.1 (2)	C8—C14—C15—C16	−167.57 (14)
C2—C1—C10—C5	0.1 (2)	C13—C14—C15—C16	−41.33 (17)
C2—C1—C10—C9	178.36 (15)	C14—C15—C16—C17	22.97 (18)
C6—C5—C10—C1	178.82 (14)	C12—C13—C17—O17	33.7 (3)
C4—C5—C10—C1	−0.9 (2)	C14—C13—C17—O17	151.02 (18)
C6—C5—C10—C9	0.5 (2)	C18—C13—C17—O17	−91.6 (2)
C4—C5—C10—C9	−179.25 (14)	C12—C13—C17—C16	−145.92 (15)
C8—C9—C10—C1	−178.42 (14)	C14—C13—C17—C16	−28.56 (16)
C11—C9—C10—C1	1.3 (2)	C18—C13—C17—C16	88.83 (16)
C8—C9—C10—C5	−0.2 (2)	C15—C16—C17—O17	−175.96 (18)
C11—C9—C10—C5	179.60 (13)	C15—C16—C17—C13	3.62 (19)
C8—C9—C11—C12	4.1 (2)		

### Hydrogen-bond geometry (Å, º)

**Table:**

*D*—H···*A*	*D*—H	H···*A*	*D*···*A*	*D*—H···*A*
O3—H3*A*···O17^i^	0.95 (3)	1.82 (3)	2.7153 (17)	157 (3)

Symmetry code: (i) −*x*+1/2, −*y*+1, *z*−1/2.
